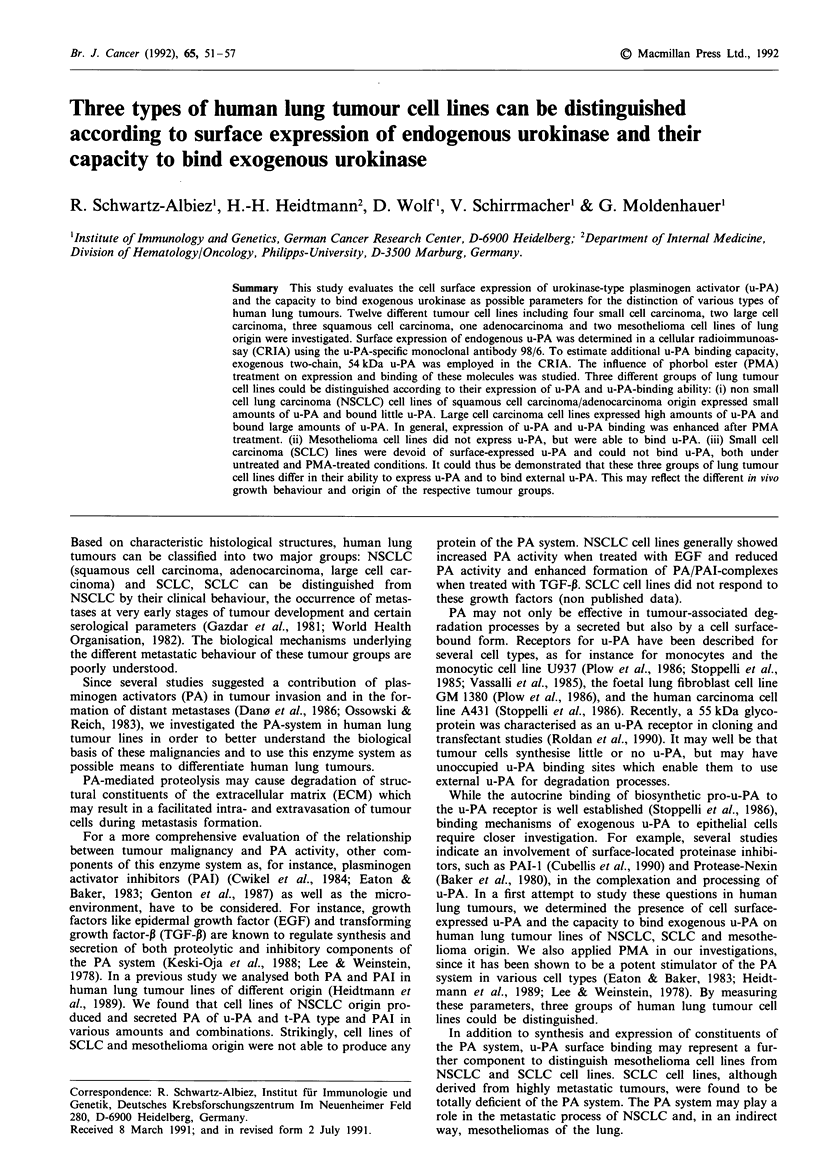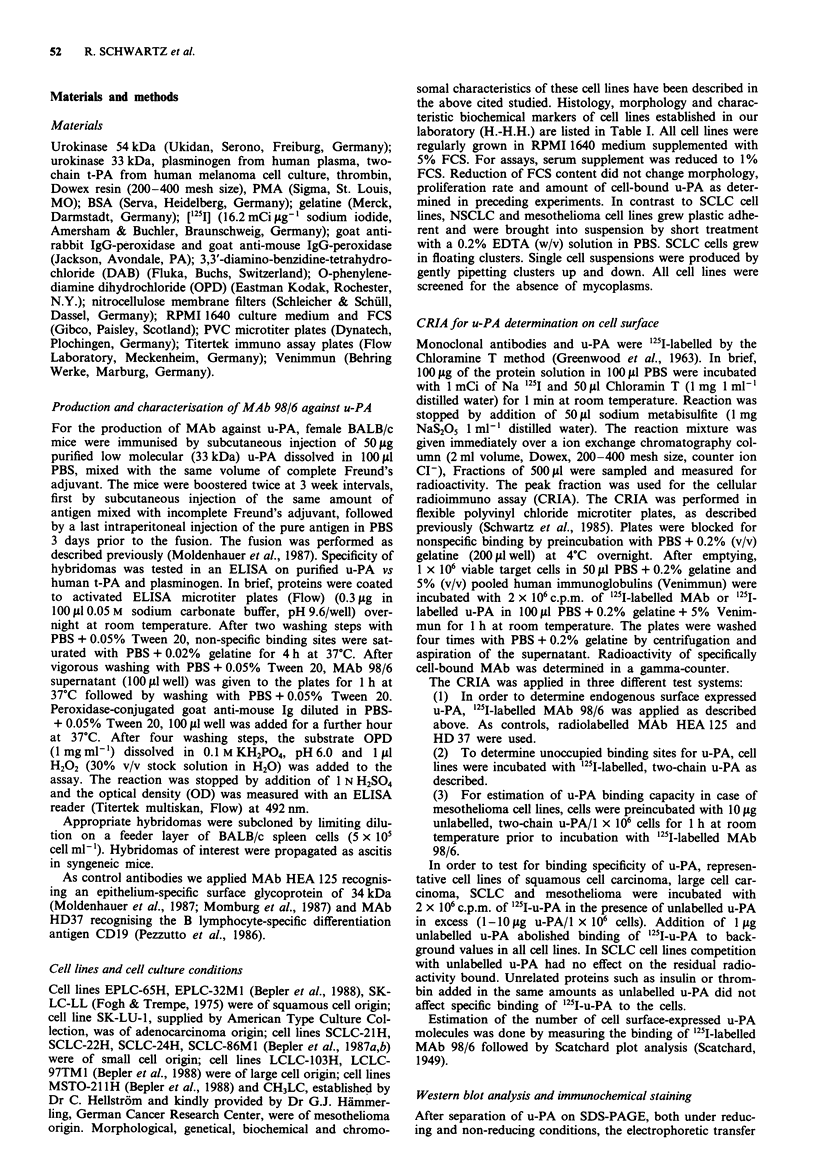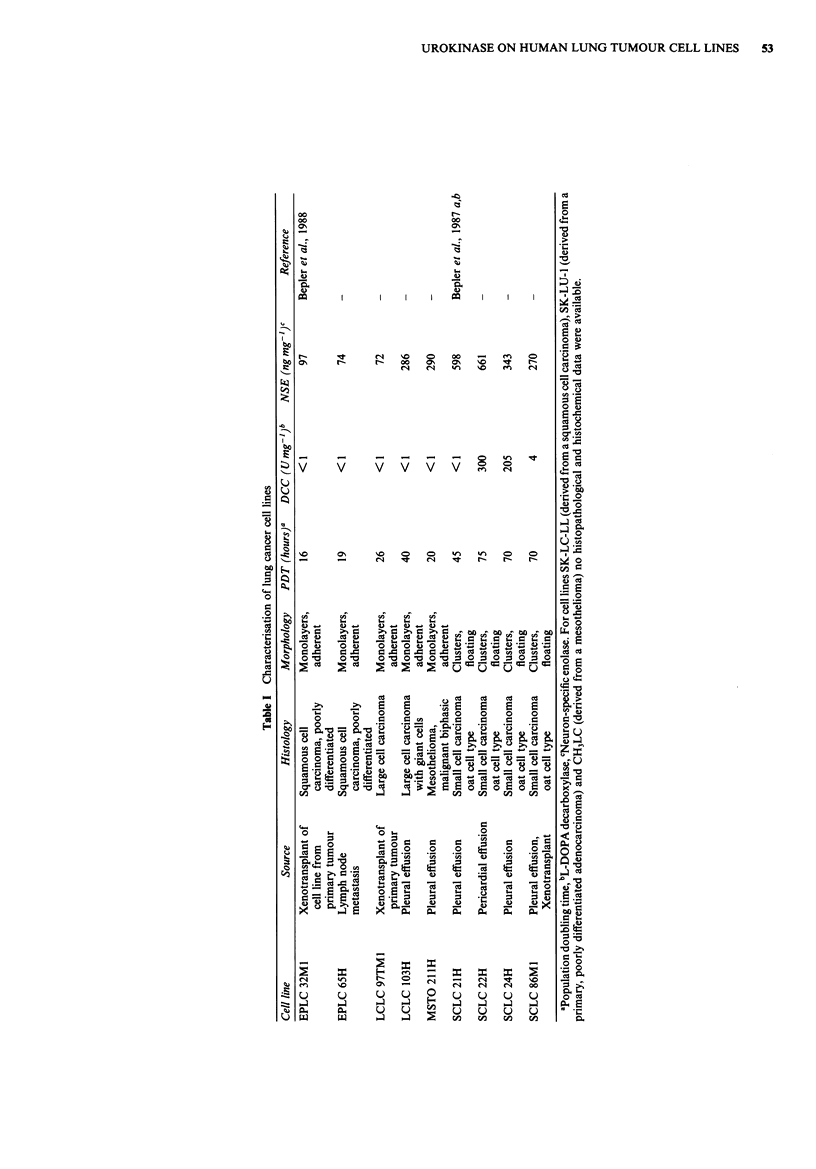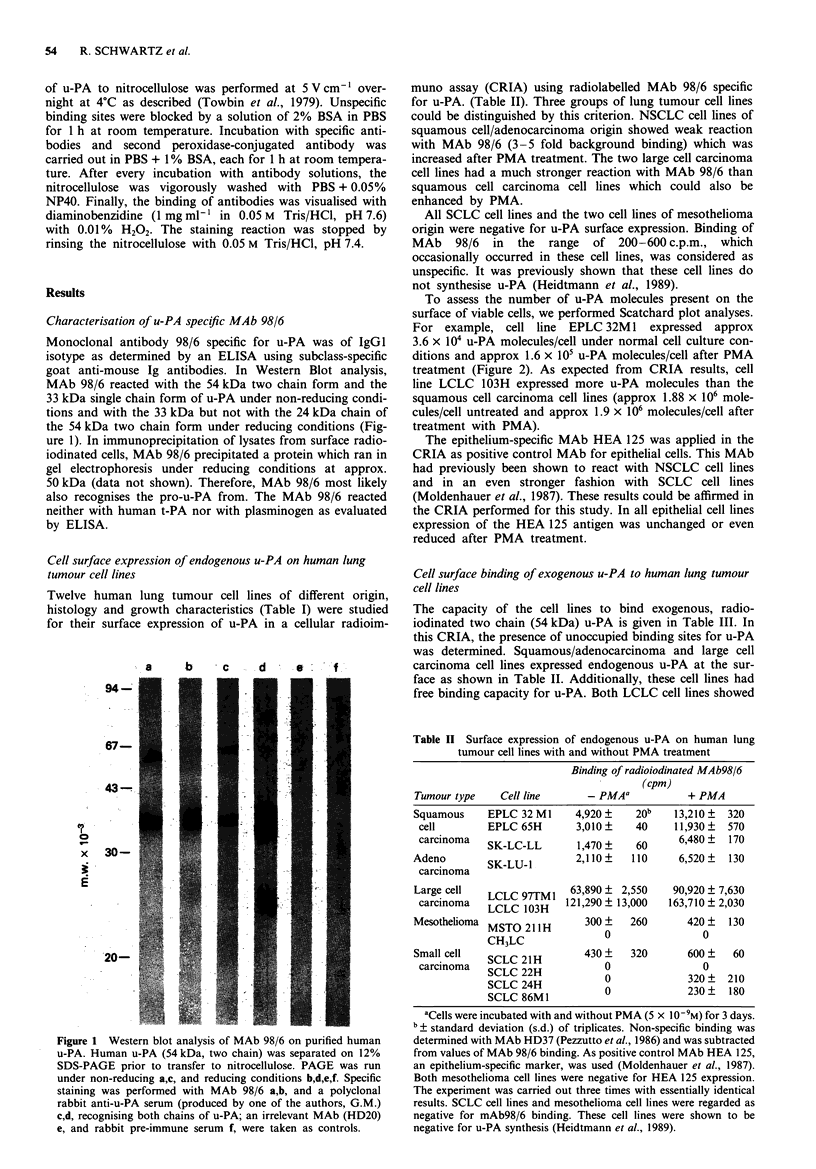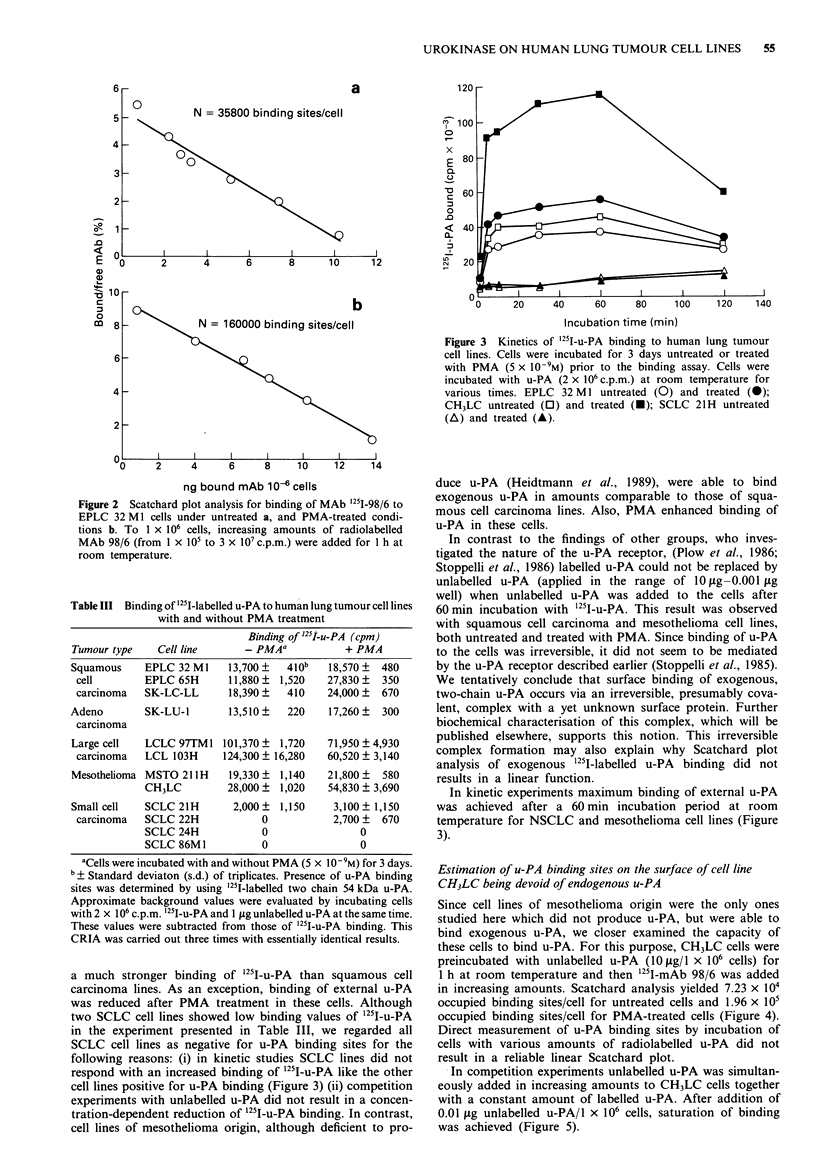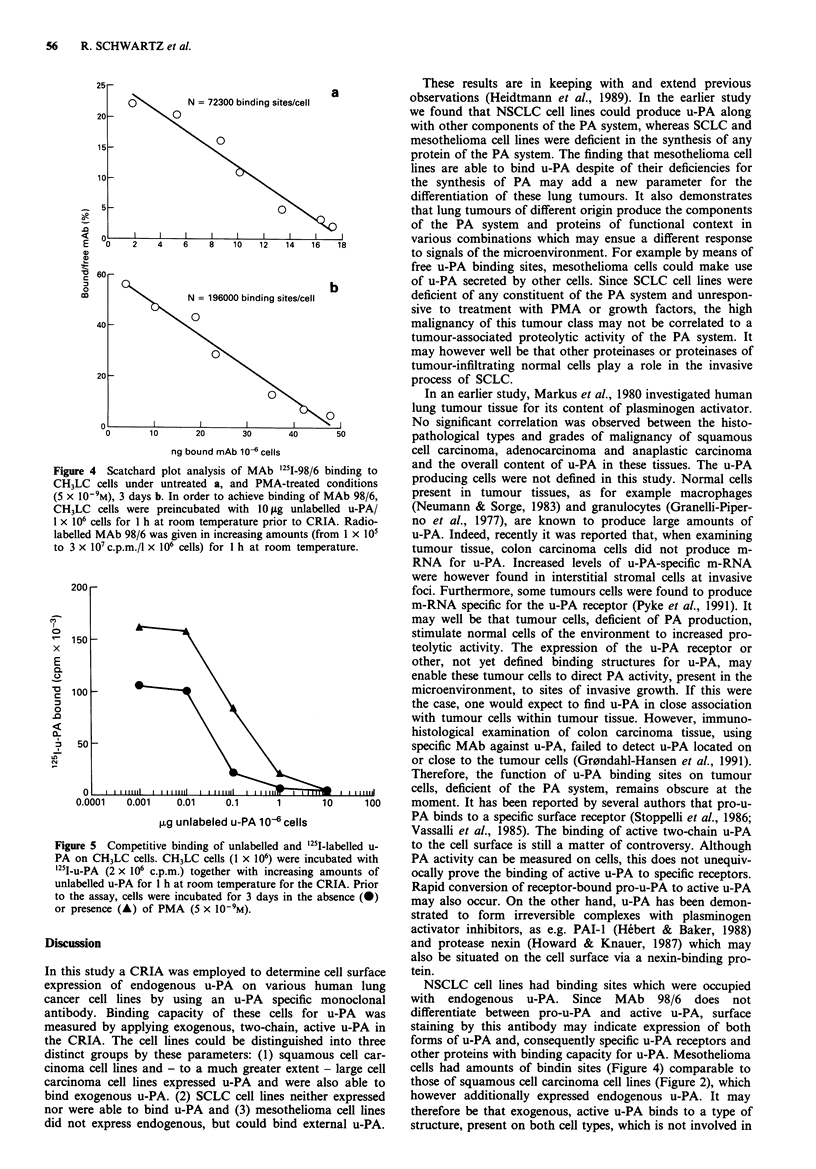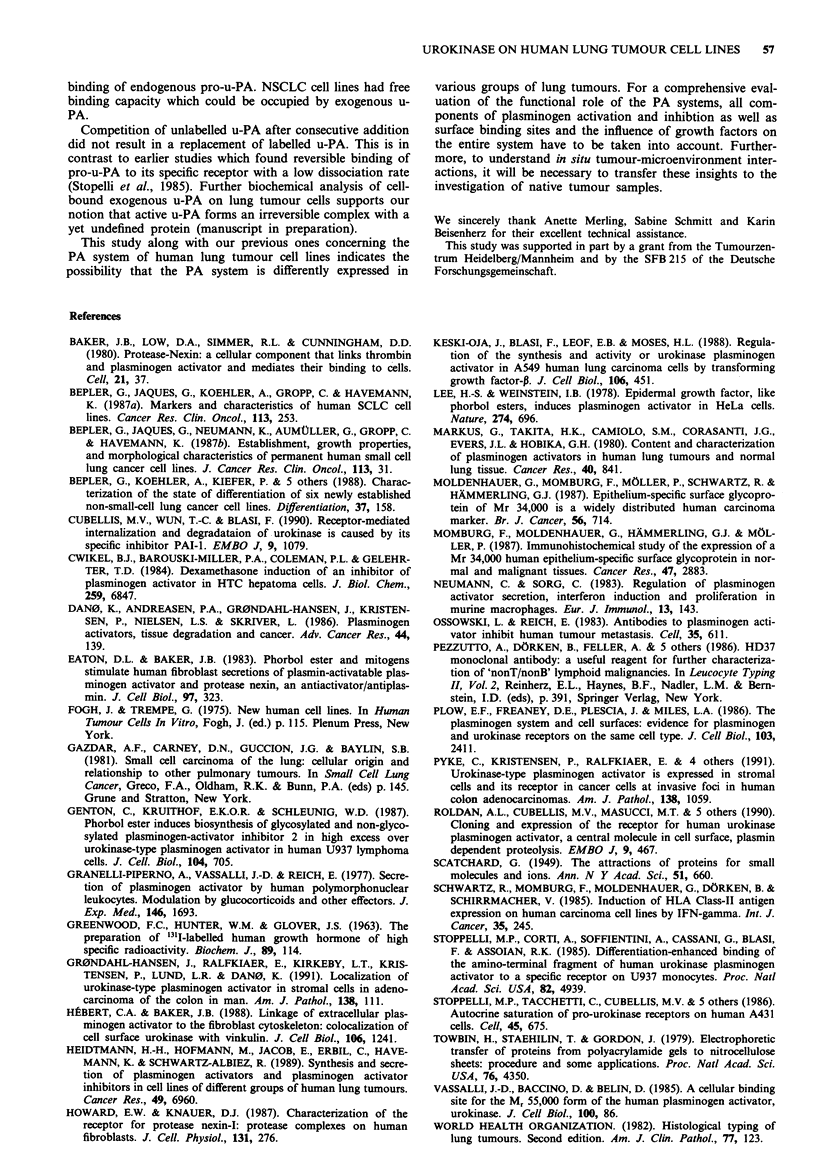# Three types of human lung tumour cell lines can be distinguished according to surface expression of endogenous urokinase and their capacity to bind exogenous urokinase.

**DOI:** 10.1038/bjc.1992.10

**Published:** 1992-01

**Authors:** R. Schwartz-Albiez, H. H. Heidtmann, D. Wolf, V. Schirrmacher, G. Moldenhauer

**Affiliations:** Institute of Immunology and Genetics, German Cancer Research Center, Heidelberg.

## Abstract

**Images:**


Br. J. Cancer (1992), 65, 51–57 © Macmillan Press Ltd., 1992

### Three types of human lung tumour cell lines can be distinguished according to surface expression of endogenous urokinase and their capacity to bind exogenous urokinase

R. Schwartz-Albiez[1], H.-H. Heidtmann[2], D. Wolf[1], V. Schirrmacher[1] & G. Moldenhauer[1]

[1]*Institute of Immunology and Genetics, German Cancer Research Center, D-6900 Heidelberg;* [2]*Department of Internal Medicine, Division of Hematology/Oncology, Philipps-University, D-3500 Marburg, Germany.*

**Summary** This study evaluates the cell surface expression of urokinase-type plasminogen activator (u-PA) and the capacity to bind exogenous urokinase as possible parameters for the distinction of various types of human lung tumours. Twelve different tumour cell lines including four small cell carcinoma, two large cell carcinoma, three squamous cell carcinoma, one adenocarcinoma and two mesothelioma cell lines of lung origin were investigated. Surface expression of endogenous u-PA was determined in a cellular radioimmunoassay (CRIA) using the u-PA-specific monoclonal antibody 98/6. To estimate additional u-PA binding capacity, exogenous two-chain, 54 kDa u-PA was employed in the CRIA. The influence of phorbol ester (PMA) treatment on expression and binding of these molecules was studied. Three different groups of lung tumour cell lines could be distinguished according to their expression of u-PA and u-PA-binding ability: (i) non small cell lung carcinoma (NSCLC) cell lines of squamous cell carcinoma/adenocarcinoma origin expressed small amounts of u-PA and bound little u-PA. Large cell carcinoma cell lines expressed high amounts of u-PA and bound large amounts of u-PA. In general, expression of u-PA and u-PA binding was enhanced after PMA treatment. (ii) Mesothelioma cell lines did not express u-PA, but were able to bind u-PA. (iii) Small cell carcinoma (SCLC) lines were devoid of surface-expressed u-PA and could not bind u-PA, both under untreated and PMA-treated conditions. It could thus be demonstrated that these three groups of lung tumour cell lines differ in their ability to express u-PA and to bind external u-PA. This may reflect the different *in vivo* growth behaviour and origin of the respective tumour groups.

Based on characteristic histological structures, human lung tumours can be classified into two major groups: NSCLC (squamous cell carcinoma, adenocarcinoma, large cell carcinoma) and SCLC, SCLC can be distinguished from NSCLC by their clinical behaviour, the occurrence of metastases at very early stages of tumour development and certain serological parameters (Gazdar *et al.*, 1981; World Health Organisation, 1982). The biological mechanisms underlying the different metastatic behaviour of these tumour groups are poorly understood.

Since several studies suggested a contribution of plasminogen activators (PA) in tumour invasion and in the formation of distant metastases (Danø *et al.*, 1986; Ossowski & Reich, 1983), we investigated the PA-system in human lung tumour lines in order to better understand the biological basis of these malignancies and to use this enzyme system as possible means to differentiate human lung tumours.

PA-mediated proteolysis may cause degradation of structural constituents of the extracellular matrix (ECM) which may result in a facilitated intra- and extravasation of tumour cells during metastasis formation.

For a more comprehensive evaluation of the relationship between tumour malignancy and PA activity, other components of this enzyme system as, for instance, plasminogen activator inhibitors (PAI) (Cwikel *et al.*, 1984; Eaton & Baker, 1983; Genton *et al.*, 1987) as well as the microenvironment, have to be considered. For instance, growth factors like epidermal growth factor (EGF) and transforming growth factor-$\beta$ (TGF-$\beta$) are known to regulate synthesis and secretion of both proteolytic and inhibitory components of the PA system (Keski-Oja *et al.*, 1988; Lee & Weinstein, 1978). In a previous study we analysed both PA and PAI in human lung tumour lines of different origin (Heidtmann *et al.*, 1989). We found that cell lines of NSCLC origin produced and secreted PA of u-PA and t-PA type and PAI in various amounts and combinations. Strikingly, cell lines of SCLC and mesothelioma origin were not able to produce any protein of the PA system. NSCLC cell lines generally showed increased PA activity when treated with EGF and reduced PA activity and enhanced formation of PA/PAI-complexes when treated with TGF-$\beta$. SCLC cell lines did not respond to these growth factors (non published data).

PA may not only be effective in tumour-associated degradation processes by a secreted but also by a cell surface-bound form. Receptors for u-PA have been described for several cell types, as for instance for monocytes and the monocytic cell line U937 (Plow *et al.*, 1986; Stoppelli *et al.*, 1985; Vassalli *et al.*, 1985), the foetal lung fibroblast cell line GM 1380 (Plow *et al.*, 1986), and the human carcinoma cell line A431 (Stoppelli *et al.*, 1986). Recently, a 55 kDa glycoprotein was characterised as an u-PA receptor in cloning and transfectant studies (Roldan *et al.*, 1990). It may well be that tumour cells synthesise little or no u-PA, but may have unoccupied u-PA binding sites which enable them to use external u-PA for degradation processes.

While the autocrine binding of biosynthetic pro-u-PA to the u-PA receptor is well established (Stoppelli *et al.*, 1986), binding mechanisms of exogenous u-PA to epithelial cells require closer investigation. For example, several studies indicate an involvement of surface-located proteinase inhibitors, such as PAI-1 (Cubellis *et al.*, 1990) and Protease-Nexin (Baker *et al.*, 1980), in the complexation and processing of u-PA. In a first attempt to study these questions in human lung tumours, we determined the presence of cell surface-expressed u-PA and the capacity to bind exogenous u-PA on human lung tumour lines of NSCLC, SCLC and mesothelioma origin. We also applied PMA in our investigations, since it has been shown to be a potent stimulator of the PA system in various cell types (Eaton & Baker, 1983; Heidtmann *et al.*, 1989; Lee & Weinstein, 1978). By measuring these parameters, three groups of human lung tumour cell lines could be distinguished.

In addition to synthesis and expression of constituents of the PA system, u-PA surface binding may represent a further component to distinguish mesothelioma cell lines from NSCLC and SCLC cell lines. SCLC cell lines, although derived from highly metastatic tumours, were found to be totally deficient of the PA system. The PA system may play a role in the metastatic process of NSCLC and, in an indirect way, mesotheliomas of the lung.

Correspondence: R. Schwartz-Albiez, Institut für Immunologie und Genetik, Deutsches Krebsforschungszentrum Im Neuenheimer Feld 280, D-6900 Heidelberg, Germany.
Received 8 March 1991; and in revised form 2 July 1991.

### Materials and methods

### *Materials*

Urokinase 54 kDa (Ukidan, Serono, Freiburg, Germany); urokinase 33 kDa, plasminogen from human plasma, two-chain t-PA from human melanoma cell culture, thrombin, Dowex resin (200–400 mesh size), PMA (Sigma, St. Louis, MO); BSA (Serva, Heidelberg, Germany); gelatine (Merck, Darmstadt, Germany); [$^{125}$I] (16.2 mCi $\mu g^{-1}$ sodium iodide, Amersham & Buchler, Braunschweig, Germany); goat anti-rabbit IgG-peroxidase and goat anti-mouse IgG-peroxidase (Jackson, Avondale, PA); 3,3′-diamino-benzidine-tetrahydrochloride (DAB) (Fluka, Buchs, Switzerland); O-phenylenediamine dihydrochloride (OPD) (Eastman Kodak, Rochester, N.Y.); nitrocellulose membrane filters (Schleicher & Schüll, Dassel, Germany); RPMI 1640 culture medium and FCS (Gibco, Paisley, Scotland); PVC microtiter plates (Dynatech, Plochingen, Germany); Titertek immuno assay plates (Flow Laboratory, Meckenheim, Germany); Venimmun (Behring Werke, Marburg, Germany).

### *Production and characterisation of MAb 98/6 against u-PA*

For the production of MAb against u-PA, female BALB/c mice were immunised by subcutaneous injection of 50 $\mu$g purified low molecular (33 kDa) u-PA dissolved in 100 $\mu$l PBS, mixed with the same volume of complete Freund's adjuvant. The mice were boostered twice at 3 week intervals, first by subcutaneous injection of the same amount of antigen mixed with incomplete Freund's adjuvant, followed by a last intraperitoneal injection of the pure antigen in PBS 3 days prior to the fusion. The fusion was performed as described previously (Moldenhauer *et al.*, 1987). Specificity of hybridomas was tested in an ELISA on purified u-PA *vs* human t-PA and plasminogen. In brief, proteins were coated to activated ELISA microtiter plates (Flow) (0.3 $\mu$g in 100 $\mu$l 0.05 M sodium carbonate buffer, pH 9.6/well) overnight at room temperature. After two washing steps with PBS + 0.05% Tween 20, non-specific binding sites were saturated with PBS + 0.02% gelatine for 4 h at 37°C. After vigorous washing with PBS + 0.05% Tween 20, MAb 98/6 supernatant (100 $\mu$l well) was given to the plates for 1 h at 37°C followed by washing with PBS + 0.05% Tween 20. Peroxidase-conjugated goat anti-mouse Ig diluted in PBS- + 0.05% Tween 20, 100 $\mu$l well was added for a further hour at 37°C. After four washing steps, the substrate OPD (1 mg ml$^{-1}$) dissolved in 0.1 M $KH_2PO_4$, pH 6.0 and 1 $\mu$l $H_2O_2$ (30% v/v stock solution in $H_2O$) was added to the assay. The reaction was stopped by addition of 1 N $H_2SO_4$ and the optical density (OD) was measured with an ELISA reader (Titertek multiskan, Flow) at 492 nm.

Appropriate hybridomas were subcloned by limiting dilution on a feeder layer of BALB/c spleen cells ($5 \times 10^5$ cell ml$^{-1}$). Hybridomas of interest were propagated as ascitis in syngeneic mice.

As control antibodies we applied MAb HEA 125 recognising an epithelium-specific surface glycoprotein of 34 kDa (Moldenhauer *et al.*, 1987; Momburg *et al.*, 1987) and MAb HD37 recognising the B lymphocyte-specific differentiation antigen CD19 (Pezzutto *et al.*, 1986).

### *Cell lines and cell culture conditions*

Cell lines EPLC-65H, EPLC-32M1 (Bepler *et al.*, 1988), SK-LC-LL (Fogh & Trempe, 1975) were of squamous cell origin; cell line SK-LU-1, supplied by American Type Culture Collection, was of adenocarcinoma origin; cell lines SCLC-21H, SCLC-22H, SCLC-24H, SCLC-86M1 (Bepler *et al.*, 1987*a*,*b*) were of small cell origin; cell lines LCLC-103H, LCLC-97TM1 (Bepler *et al.*, 1988) were of large cell origin; cell lines MSTO-211H (Bepler *et al.*, 1988) and $CH_3LC$, established by Dr C. Hellström and kindly provided by Dr G.J. Hämmerling, German Cancer Research Center, were of mesothelioma origin. Morphological, genetical, biochemical and chromosomal characteristics of these cell lines have been described in the above cited studied. Histology, morphology and characteristic biochemical markers of cell lines established in our laboratory (H.-H.H.) are listed in Table I. All cell lines were regularly grown in RPMI 1640 medium supplemented with 5% FCS. For assays, serum supplement was reduced to 1% FCS. Reduction of FCS content did not change morphology, proliferation rate and amount of cell-bound u-PA as determined in preceding experiments. In contrast to SCLC cell lines, NSCLC and mesothelioma cell lines grew plastic adherent and were brought into suspension by short treatment with a 0.2% EDTA (w/v) solution in PBS. SCLC cells grew in floating clusters. Single cell suspensions were produced by gently pipetting clusters up and down. All cell lines were screened for the absence of mycoplasms.

### *CRIA for u-PA determination on cell surface*

Monoclonal antibodies and u-PA were $^{125}$I-labelled by the Chloramine T method (Greenwood *et al.*, 1963). In brief, 100 $\mu$g of the protein solution in 100 $\mu$l PBS were incubated with 1 mCi of Na $^{125}$I and 50 $\mu$l Chloramin T (1 mg 1 ml$^{-1}$ distilled water) for 1 min at room temperature. Reaction was stopped by addition of 50 $\mu$l sodium metabisulfite (1 mg $NaS_2O_5$ 1 ml$^{-1}$ distilled water). The reaction mixture was given immediately over a ion exchange chromatography column (2 ml volume, Dowex, 200–400 mesh size, counter ion $Cl^-$), Fractions of 500 $\mu$l were sampled and measured for radioactivity. The peak fraction was used for the cellular radioimmuno assay (CRIA). The CRIA was performed in flexible polyvinyl chloride microtiter plates, as described previously (Schwartz *et al.*, 1985). Plates were blocked for nonspecific binding by preincubation with PBS + 0.2% (v/v) gelatine (200 $\mu$l well) at 4°C overnight. After emptying, $1 \times 10^6$ viable target cells in 50 $\mu$l PBS + 0.2% gelatine and 5% (v/v) pooled human immunoglobulins (Venimmun) were incubated with $2 \times 10^6$ c.p.m. of $^{125}$I-labelled MAb or $^{125}$I-labelled u-PA in 100 $\mu$l PBS + 0.2% gelatine + 5% Venimmun for 1 h at room temperature. The plates were washed four times with PBS + 0.2% gelatine by centrifugation and aspiration of the supernatant. Radioactivity of specifically cell-bound MAb was determined in a gamma-counter.

The CRIA was applied in three different test systems:

(1) In order to determine endogenous surface expressed u-PA, $^{125}$I-labelled MAb 98/6 was applied as described above. As controls, radiolabelled MAb HEA 125 and HD 37 were used.

(2) To determine unoccupied binding sites for u-PA, cell lines were incubated with $^{125}$I-labelled, two-chain u-PA as described.

(3) For estimation of u-PA binding capacity in case of mesothelioma cell lines, cells were preincubated with 10 $\mu$g unlabelled, two-chain u-PA/1 × 10$^6$ cells for 1 h at room temperature prior to incubation with $^{125}$I-labelled MAb 98/6.

In order to test for binding specificity of u-PA, representative cell lines of squamous cell carcinoma, large cell carcinoma, SCLC and mesothelioma were incubated with $2 \times 10^6$ c.p.m. of $^{125}$I-u-PA in the presence of unlabelled u-PA in excess (1–10 $\mu$g u-PA/1 × 10$^6$ cells). Addition of 1 $\mu$g unlabelled u-PA abolished binding of $^{125}$I-u-PA to background values in all cell lines. In SCLC cell lines competition with unlabelled u-PA had no effect on the residual radioactivity bound. Unrelated proteins such as insulin or thrombin added in the same amounts as unlabelled u-PA did not affect specific binding of $^{125}$I-u-PA to the cells.

Estimation of the number of cell surface-expressed u-PA molecules was done by measuring the binding of $^{125}$I-labelled MAb 98/6 followed by Scatchard plot analysis (Scatchard, 1949).

### *Western blot analysis and immunochemical staining*

After separation of u-PA on SDS-PAGE, both under reducing and non-reducing conditions, the electrophoretic transfer

**Table I** Characterisation of lung cancer cell lines

| *Cell line* | *Source* | *Histology* | *Morphology* | *PDT (hours)*[a] | *DCC* $(U\ mg^{-1})^b$ | *NSE* $(ng\ mg^{-1})^c$ | *Reference* |
|---|---|---|---|---|---|---|---|
| EPLC 32M1 | Xenotransplant of cell line from primary tumour | Squamous cell carcinoma, poorly differentiated | Monolayers, adherent | 16 | <1 | 97 | Bepler *et al.*, 1988 |
| EPLC 65H | Lymph node metastasis | Squamous cell carcinoma, poorly differentiated | Monolayers, adherent | 19 | <1 | 74 | – |
| LCLC 97TM1 | Xenotransplant of primary tumour | Large cell carcinoma | Monolayers, adherent | 26 | <1 | 72 | – |
| LCLC 103H | Pleural effusion | Large cell carcinoma with giant cells | Monolayers, adherent | 40 | <1 | 286 | – |
| MSTO 211H | Pleural effusion | Mesothelioma, malignant biphasic | Monolayers, adherent | 20 | <1 | 290 | – |
| SCLC 21H | Pleural effusion | Small cell carcinoma oat cell type | Clusters, floating | 45 | <1 | 598 | Bepler *et al.*, 1987 *a,b* |
| SCLC 22H | Pericardial effusion | Small cell carcinoma oat cell type | Clusters, floating | 75 | 300 | 661 | – |
| SCLC 24H | Pleural effusion | Small cell carcinoma oat cell type | Clusters, floating | 70 | 205 | 343 | – |
| SCLC 86M1 | Pleural effusion, Xenotransplant | Small cell carcinoma oat cell type | Clusters, floating | 70 | 4 | 270 | – |

[a]Population doubling time, [b]L-DOPA decarboxylase, [c]Neuron-specific enolase. For cell lines SK-LC-LL (derived from a squamous cell carcinoma), SK-LU-1 (derived from a primary, poorly differentiated adenocarcinoma) and $CH_3LC$ (derived from a mesothelioma) no histopathological and histochemical data were available.

of u-PA to nitrocellulose was performed at $5\ V\ cm^{-1}$ overnight at 4°C as described (Towbin *et al.*, 1979). Unspecific binding sites were blocked by a solution of 2% BSA in PBS for 1 h at room temperature. Incubation with specific antibodies and second peroxidase-conjugated antibody was carried out in PBS + 1% BSA, each for 1 h at room temperature. After every incubation with antibody solutions, the nitrocellulose was vigorously washed with PBS + 0.05% NP40. Finally, the binding of antibodies was visualised with diaminobenzidine ($1\ mg\ ml^{-1}$ in 0.05 M Tris/HCl, pH 7.6) with 0.01% $H_2O_2$. The staining reaction was stopped by rinsing the nitrocellulose with 0.05 M Tris/HCl, pH 7.4.

### Results

### *Characterisation of u-PA specific MAb 98/6*

Monoclonal antibody 98/6 specific for u-PA was of IgG1 isotype as determined by an ELISA using subclass-specific goat anti-mouse Ig antibodies. In Western Blot analysis, MAb 98/6 reacted with the 54 kDa two chain form and the 33 kDa single chain form of u-PA under non-reducing conditions and with the 33 kDa but not with the 24 kDa chain of the 54 kDa two chain form under reducing conditions (Figure 1). In immunoprecipitation of lysates from surface radioiodinated cells, MAb 98/6 precipitated a protein which ran in gel electrophoresis under reducing conditions at approx. 50 kDa (data not shown). Therefore, MAb 98/6 most likely also recognises the pro-u-PA from. The MAb 98/6 reacted neither with human t-PA nor with plasminogen as evaluated by ELISA.

### *Cell surface expression of endogenous u-PA on human lung tumour cell lines*

Twelve human lung tumour cell lines of different origin, histology and growth characteristics (Table I) were studied for their surface expression of u-PA in a cellular radioimmuno assay (CRIA) using radiolabelled MAb 98/6 specific for u-PA. (Table II). Three groups of lung tumour cell lines could be distinguished by this criterion. NSCLC cell lines of squamous cell/adenocarcinoma origin showed weak reaction with MAb 98/6 (3–5 fold background binding) which was increased after PMA treatment. The two large cell carcinoma cell lines had a much stronger reaction with MAb 98/6 than squamous cell carcinoma cell lines which could also be enhanced by PMA.

**Figure 1** Western blot analysis of MAb 98/6 on purified human u-PA. Human u-PA (54 kDa, two chain) was separated on 12% SDS-PAGE prior to transfer to nitrocellulose. PAGE was run under non-reducing **a**,**c**, and reducing conditions **b**,**d**,**e**,**f**. Specific staining was performed with MAb 98/6 **a**,**b**, and a polyclonal rabbit anti-u-PA serum (produced by one of the authors, G.M.) **c**,**d**, recognising both chains of u-PA; an irrelevant MAb (HD20) **e**, and rabbit pre-immune serum **f**, were taken as controls.

All SCLC cell lines and the two cell lines of mesothelioma origin were negative for u-PA surface expression. Binding of MAb 98/6 in the range of 200–600 c.p.m., which occasionally occurred in these cell lines, was considered as unspecific. It was previously shown that these cell lines do not synthesise u-PA (Heidtmann *et al.*, 1989).

To assess the number of u-PA molecules present on the surface of viable cells, we performed Scatchard plot analyses. For example, cell line EPLC 32M1 expressed approx $3.6 \times 10^4$ u-PA molecules/cell under normal cell culture conditions and approx $1.6 \times 10^5$ u-PA molecules/cell after PMA treatment (Figure 2). As expected from CRIA results, cell line LCLC 103H expressed more u-PA molecules than the squamous cell carcinoma cell lines (approx $1.88 \times 10^6$ molecules/cell untreated and approx $1.9 \times 10^6$ molecules/cell after treatment with PMA).

The epithelium-specific MAb HEA 125 was applied in the CRIA as positive control MAb for epithelial cells. This MAb had previously been shown to react with NSCLC cell lines and in an even stronger fashion with SCLC cell lines (Moldenhauer *et al.*, 1987). These results could be affirmed in the CRIA performed for this study. In all epithelial cell lines expression of the HEA 125 antigen was unchanged or even reduced after PMA treatment.

### *Cell surface binding of exogenous u-PA to human lung tumour cell lines*

The capacity of the cell lines to bind exogenous, radioiodinated two chain (54 kDa) u-PA is given in Table III. In this CRIA, the presence of unoccupied binding sites for u-PA was determined. Squamous/adenocarcinoma and large cell carcinoma cell lines expressed endogenous u-PA at the surface as shown in Table II. Additionally, these cell lines had free binding capacity for u-PA. Both LCLC cell lines showed

**Table II** Surface expression of endogenous u-PA on human lung tumour cell lines with and without PMA treatment

| *Tumour type* | *Cell line* | *Binding of radioiodinated MAb98/6 (cpm)* – *PMA*[a] | + *PMA* |
|---|---|---|---|
| Squamous cell carcinoma | EPLC 32 M1 | 4,920 ± 20[b] | 13,210 ± 320 |
| | EPLC 65H | 3,010 ± 40 | 11,930 ± 570 |
| | SK-LC-LL | 1,470 ± 60 | 6,480 ± 170 |
| Adeno carcinoma | SK-LU-1 | 2,110 ± 110 | 6,520 ± 130 |
| Large cell carcinoma | LCLC 97TM1 | 63,890 ± 2,550 | 90,920 ± 7,630 |
| | LCLC 103H | 121,290 ± 13,000 | 163,710 ± 2,030 |
| Mesothelioma | MSTO 211H | 300 ± 260 | 420 ± 130 |
| | $CH_3LC$ | 0 | 0 |
| Small cell carcinoma | SCLC 21H | 430 ± 320 | 600 ± 60 |
| | SCLC 22H | 0 | 0 |
| | SCLC 24H | 0 | 320 ± 210 |
| | SCLC 86M1 | 0 | 230 ± 180 |

[a]Cells were incubated with and without PMA ($5 \times 10^{-9}$M) for 3 days. [b]± standard deviation (s.d.) of triplicates. Non-specific binding was determined with MAb HD37 (Pezzutto *et al.*, 1986) and was subtracted from values of MAb 98/6 binding. As positive control MAb HEA 125, an epithelium-specific marker, was used (Moldenhauer *et al.*, 1987). Both mesothelioma cell lines were negative for HEA 125 expression. The experiment was carried out three times with essentially identical results. SCLC cell lines and mesothelioma cell lines were regarded as negative for mAb98/6 binding. These cell lines were shown to be negative for u-PA synthesis (Heidtmann *et al.*, 1989).

**Figure 2** Scatchard plot analysis for binding of MAb $^{125}$I-98/6 to EPLC 32 M1 cells under untreated **a**, and PMA-treated conditions **b**. To $1 \times 10^6$ cells, increasing amounts of radiolabelled MAb 98/6 (from $1 \times 10^5$ to $3 \times 10^7$ c.p.m.) were added for 1 h at room temperature.

**Table III** Binding of $^{125}$I-labelled u-PA to human lung tumour cell lines with and without PMA treatment

| Tumour type | Cell line | Binding of $^{125}$I-u-PA (cpm) − PMA[a] | + PMA |
|---|---|---|---|
| Squamous | EPLC 32 M1 | 13,700 ± 410[b] | 18,570 ± 480 |
| cell | EPLC 65H | 11,880 ± 1,520 | 27,830 ± 350 |
| carcinoma | SK-LC-LL | 18,390 ± 410 | 24,000 ± 670 |
| Adeno carcinoma | SK-LU-1 | 13,510 ± 220 | 17,260 ± 300 |
| Large cell | LCLC 97TM1 | 101,370 ± 1,720 | 71,950 ± 4,930 |
| carcinoma | LCL 103H | 124,300 ± 16,280 | 60,520 ± 3,140 |
| Mesothelioma | MSTO 211H | 19,330 ± 1,140 | 21,800 ± 580 |
| | $CH_3LC$ | 28,000 ± 1,020 | 54,830 ± 3,690 |
| Small cell | SCLC 21H | 2,000 ± 1,150 | 3,100 ± 1,150 |
| carcinoma | SCLC 22H | 0 | 2,700 ± 670 |
| | SCLC 24H | 0 | 0 |
| | SCLC 86M1 | 0 | 0 |

[a]Cells were incubated with and without PMA ($5 \times 10^{-9}$M) for 3 days. [b]± Standard deviaton (s.d.) of triplicates. Presence of u-PA binding sites was determined by using $^{125}$I-labelled two chain 54 kDa u-PA. Approximate background values were evaluated by incubating cells with $2 \times 10^6$ c.p.m. $^{125}$I-u-PA and 1 µg unlabelled u-PA at the same time. These values were subtracted from those of $^{125}$I-u-PA binding. This CRIA was carried out three times with essentially identical results.

a much stronger binding of $^{125}$I-u-PA than squamous cell carcinoma lines. As an exception, binding of external u-PA was reduced after PMA treatment in these cells. Although two SCLC cell lines showed low binding values of $^{125}$I-u-PA in the experiment presented in Table III, we regarded all SCLC cell lines as negative for u-PA binding sites for the following reasons: (i) in kinetic studies SCLC lines did not respond with an increased binding of $^{125}$I-u-PA like the other cell lines positive for u-PA binding (Figure 3) (ii) competition experiments with unlabelled u-PA did not result in a concentration-dependent reduction of $^{125}$I-u-PA binding. In contrast, cell lines of mesothelioma origin, although deficient to produce u-PA (Heidtmann *et al.*, 1989), were able to bind exogenous u-PA in amounts comparable to those of squamous cell carcinoma lines. Also, PMA enhanced binding of u-PA in these cells.

**Figure 3** Kinetics of $^{125}$I-u-PA binding to human lung tumour cell lines. Cells were incubated for 3 days untreated or treated with PMA ($5 \times 10^{-9}$M) prior to the binding assay. Cells were incubated with u-PA ($2 \times 10^6$ c.p.m.) at room temperature for various times. EPLC 32 M1 untreated (○) and treated (●); $CH_3LC$ untreated (□) and treated (■); SCLC 21H untreated (△) and treated (▲).

In contrast to the findings of other groups, who investigated the nature of the u-PA receptor, (Plow *et al.*, 1986; Stoppelli *et al.*, 1986) labelled u-PA could not be replaced by unlabelled u-PA (applied in the range of 10 µg–0.001 µg well) when unlabelled u-PA was added to the cells after 60 min incubation with $^{125}$I-u-PA. This result was observed with squamous cell carcinoma and mesothelioma cell lines, both untreated and treated with PMA. Since binding of u-PA to the cells was irreversible, it did not seem to be mediated by the u-PA receptor described earlier (Stoppelli *et al.*, 1985). We tentatively conclude that surface binding of exogenous, two-chain u-PA occurs via an irreversible, presumably covalent, complex with a yet unknown surface protein. Further biochemical characterisation of this complex, which will be published elsewhere, supports this notion. This irreversible complex formation may also explain why Scatchard plot analysis of exogenous $^{125}$I-labelled u-PA binding did not results in a linear function.

In kinetic experiments maximum binding of external u-PA was achieved after a 60 min incubation period at room temperature for NSCLC and mesothelioma cell lines (Figure 3).

### *Estimation of u-PA binding sites on the surface of cell line $CH_3LC$ being devoid of endogenous u-PA*

Since cell lines of mesothelioma origin were the only ones studied here which did not produce u-PA, but were able to bind exogenous u-PA, we closer examined the capacity of these cells to bind u-PA. For this purpose, $CH_3LC$ cells were preincubated with unlabelled u-PA (10 µg/l $\times 10^6$ cells) for 1 h at room temperature and then $^{125}$I-mAb 98/6 was added in increasing amounts. Scatchard analysis yielded $7.23 \times 10^4$ occupied binding sites/cell for untreated cells and $1.96 \times 10^5$ occupied binding sites/cell for PMA-treated cells (Figure 4). Direct measurement of u-PA binding sites by incubation of cells with various amounts of radiolabelled u-PA did not result in a reliable linear Scatchard plot.

In competition experiments unlabelled u-PA was simultaneously added in increasing amounts to $CH_3LC$ cells together with a constant amount of labelled u-PA. After addition of 0.01 µg unlabelled u-PA/1 $\times 10^6$ cells, saturation of binding was achieved (Figure 5).

**Figure 4** Scatchard plot analysis of MAb $^{125}$I-98/6 binding to $CH_3LC$ cells under untreated **a**, and PMA-treated conditions ($5 \times 10^{-9}$M), 3 days **b**. In order to achieve binding of MAb 98/6, $CH_3LC$ cells were preincubated with 10 μg unlabelled u-PA/ $1 \times 10^6$ cells for 1 h at room temperature prior to CRIA. Radiolabelled MAb 98/6 was given in increasing amounts (from $1 \times 10^5$ to $3 \times 10^7$ c.p.m./l $\times 10^6$ cells) for 1 h at room temperature.

**Figure 5** Competitive binding of unlabelled and $^{125}$I-labelled u-PA on $CH_3LC$ cells. $CH_3LC$ cells ($1 \times 10^6$) were incubated with $^{125}$I-u-PA ($2 \times 10^6$ c.p.m.) together with increasing amounts of unlabelled u-PA for 1 h at room temperature for the CRIA. Prior to the assay, cells were incubated for 3 days in the absence (●) or presence (▲) of PMA ($5 \times 10^{-9}$M).

### Discussion

In this study a CRIA was employed to determine cell surface expression of endogenous u-PA on various human lung cancer cell lines by using an u-PA specific monoclonal antibody. Binding capacity of these cells for u-PA was measured by applying exogenous, two-chain, active u-PA in the CRIA. The cell lines could be distinguished into three distinct groups by these parameters: (1) squamous cell carcinoma cell lines and – to a much greater extent – large cell carcinoma cell lines expressed u-PA and were also able to bind exogenous u-PA. (2) SCLC cell lines neither expressed nor were able to bind u-PA and (3) mesothelioma cell lines did not express endogenous, but could bind external u-PA.

These results are in keeping with and extend previous observations (Heidtmann *et al.*, 1989). In the earlier study we found that NSCLC cell lines could produce u-PA along with other components of the PA system, whereas SCLC and mesothelioma cell lines were deficient in the synthesis of any protein of the PA system. The finding that mesothelioma cell lines are able to bind u-PA despite of their deficiencies for the synthesis of PA may add a new parameter for the differentiation of these lung tumours. It also demonstrates that lung tumours of different origin produce the components of the PA system and proteins of functional context in various combinations which may ensue a different response to signals of the microenvironment. For example by means of free u-PA binding sites, mesothelioma cells could make use of u-PA secreted by other cells. Since SCLC cell lines were deficient of any constituent of the PA system and unresponsive to treatment with PMA or growth factors, the high malignancy of this tumour class may not be correlated to a tumour-associated proteolytic activity of the PA system. It may however well be that other proteinases or proteinases of tumour-infiltrating normal cells play a role in the invasive process of SCLC.

In an earlier study, Markus *et al.*, 1980 investigated human lung tumour tissue for its content of plasminogen activator. No significant correlation was observed between the histopathological types and grades of malignancy of squamous cell carcinoma, adenocarcinoma and anaplastic carcinoma and the overall content of u-PA in these tissues. The u-PA producing cells were not defined in this study. Normal cells present in tumour tissues, as for example macrophages (Neumann & Sorge, 1983) and granulocytes (Granelli-Piperno *et al.*, 1977), are known to produce large amounts of u-PA. Indeed, recently it was reported that, when examining tumour tissue, colon carcinoma cells did not produce m-RNA for u-PA. Increased levels of u-PA-specific m-RNA were however found in interstitial stromal cells at invasive foci. Furthermore, some tumours cells were found to produce m-RNA specific for the u-PA receptor (Pyke *et al.*, 1991). It may well be that tumour cells, deficient of PA production, stimulate normal cells of the environment to increased proteolytic activity. The expression of the u-PA receptor or other, not yet defined binding structures for u-PA, may enable these tumour cells to direct PA activity, present in the microenvironment, to sites of invasive growth. If this were the case, one would expect to find u-PA in close association with tumour cells within tumour tissue. However, immunohistological examination of colon carcinoma tissue, using specific MAb against u-PA, failed to detect u-PA located on or close to the tumour cells (Grøndahl-Hansen *et al.*, 1991). Therefore, the function of u-PA binding sites on tumour cells, deficient of the PA system, remains obscure at the moment. It has been reported by several authors that pro-u-PA binds to a specific surface receptor (Stoppelli *et al.*, 1986; Vassalli *et al.*, 1985). The binding of active two-chain u-PA to the cell surface is still a matter of controversy. Although PA activity can be measured on cells, this does not unequivocally prove the binding of active u-PA to specific receptors. Rapid conversion of receptor-bound pro-u-PA to active u-PA may also occur. On the other hand, u-PA has been demonstrated to form irreversible complexes with plasminogen activator inhibitors, as e.g. PAI-1 (Hébert & Baker, 1988) and protease nexin (Howard & Knauer, 1987) which may also be situated on the cell surface via a nexin-binding protein.

NSCLC cell lines had binding sites which were occupied with endogenous u-PA. Since MAb 98/6 does not differentiate between pro-u-PA and active u-PA, surface staining by this antibody may indicate expression of both forms of u-PA and, consequently specific u-PA receptors and other proteins with binding capacity for u-PA. Mesothelioma cells had amounts of bindin sites (Figure 4) comparable to those of squamous cell carcinoma cell lines (Figure 2), which however additionally expressed endogenous u-PA. It may therefore be that exogenous, active u-PA binds to a type of structure, present on both cell types, which is not involved in binding of endogenous pro-u-PA. NSCLC cell lines had free binding capacity which could be occupied by exogenous u-PA.

Competition of unlabelled u-PA after consecutive addition did not result in a replacement of labelled u-PA. This is in contrast to earlier studies which found reversible binding of pro-u-PA to its specific receptor with a low dissociation rate (Stopelli *et al.*, 1985). Further biochemical analysis of cell-bound exogenous u-PA on lung tumour cells supports our notion that active u-PA forms an irreversible complex with a yet undefined protein (manuscript in preparation).

This study along with our previous ones concerning the PA system of human lung tumour cell lines indicates the possibility that the PA system is differently expressed in various groups of lung tumours. For a comprehensive evaluation of the functional role of the PA systems, all components of plasminogen activation and inhibtion as well as surface binding sites and the influence of growth factors on the entire system have to be taken into account. Furthermore, to understand *in situ* tumour-microenvironment interactions, it will be necessary to transfer these insights to the investigation of native tumour samples.

We sincerely thank Anette Merling, Sabine Schmitt and Karin Beisenherz for their excellent technical assistance.

This study was supported in part by a grant from the Tumourzentrum Heidelberg/Mannheim and by the SFB 215 of the Deutsche Forschungsgemeinschaft.

### References

BAKER, J.B., LOW, D.A., SIMMER, R.L. & CUNNINGHAM, D.D. (1980). Protease-Nexin: a cellular component that links thrombin and plasminogen activator and mediates their binding to cells. *Cell*, **21**, 37.

BEPLER, G., JAQUES, G., KOEHLER, A., GROPP, C. & HAVEMANN, K. (1987*a*). Markers and characteristics of human SCLC cell lines. *Cancer Res. Clin. Oncol.*, **113**, 253.

BEPLER, G., JAQUES, G., NEUMANN, K., AUMÜLLER, G., GROPP, C. & HAVEMANN, K. (1987*b*). Establishment, growth properties, and morphological characteristics of permanent human small cell lung cancer cell lines. *J. Cancer Res. Clin. Oncol.*, **113**, 31.

BEPLER, G., KOEHLER, A., KIEFER, P. & 5 others (1988). Characterization of the state of differentiation of six newly established non-small-cell lung cancer cell lines. *Differentiation*, **37**, 158.

CUBELLIS, M.V., WUN, T.-C. & BLASI, F. (1990). Receptor-mediated internalization and degradataion of urokinase is caused by its specific inhibitor PAI-1. *EMBO J*, **9**, 1079.

CWIKEL, B.J., BAROUSKI-MILLER, P.A., COLEMAN, P.L. & GELEHRTER, T.D. (1984). Dexamethasone induction of an inhibitor of plasminogen activator in HTC hepatoma cells. *J. Biol. Chem.*, **259**, 6847.

DANØ, K., ANDREASEN, P.A., GRØNDAHL-HANSEN, J., KRISTENSEN, P., NIELSEN, L.S. & SKRIVER, L. (1986). Plasminogen activators, tissue degradation and cancer. *Adv. Cancer Res.*, **44**, 139.

EATON, D.L. & BAKER, J.B. (1983). Phorbol ester and mitogens stimulate human fibroblast secretions of plasmin-activatable plasminogen activator and protease nexin, an antiactivator/antiplasmin. *J. Cell Biol.*, **97**, 323.

FOGH, J. & TREMPE, G. (1975). New human cell lines. In *Human Tumour Cells In Vitro*, Fogh, J. (ed.) p. 115. Plenum Press, New York.

GAZDAR, A.F., CARNEY, D.N., GUCCION, J.G. & BAYLIN, S.B. (1981). Small cell carcinoma of the lung: cellular origin and relationship to other pulmonary tumours. In *Small Cell Lung Cancer*, Greco, F.A., Oldham, R.K. & Bunn, P.A. (eds) p. 145. Grune and Stratton, New York.

GENTON, C., KRUITHOF, E.K.O.R. & SCHLEUNIG, W.D. (1987). Phorbol ester induces biosynthesis of glycosylated and non-glycosylated plasminogen-activator inhibitor 2 in high excess over urokinase-type plasminogen activator in human U937 lymphoma cells. *J. Cell. Biol.*, **104**, 705.

GRANELLI-PIPERNO, A., VASSALLI, J.-D. & REICH, E. (1977). Secretion of plasminogen activator by human polymorphonuclear leukocytes. Modulation by glucocorticoids and other effectors. *J. Exp. Med.*, **146**, 1693.

GREENWOOD, F.C., HUNTER, W.M. & GLOVER, J.S. (1963). The preparation of $^{131}$I-labelled human growth hormone of high specific radioactivity. *Biochem. J.*, **89**, 114.

GRØNDAHL-HANSEN, J., RALFKIAER, E., KIRKEBY, L.T., KRISTENSEN, P., LUND, L.R. & DANØ, K. (1991). Localization of urokinase-type plasminogen activator in stromal cells in adenocarcinoma of the colon in man. *Am. J. Pathol.*, **138**, 111.

HÉBERT, C.A. & BAKER, J.B. (1988). Linkage of extracellular plasminogen activator to the fibroblast cytoskeleton: colocalization of cell surface urokinase with vinkulin. *J. Cell Biol.*, **106**, 1241.

HEIDTMANN, H.-H., HOFMANN, M., JACOB, E., ERBIL, C., HAVEMANN, K. & SCHWARTZ-ALBIEZ, R. (1989). Synthesis and secretion of plasminogen activators and plasminogen activator inhibitors in cell lines of different groups of human lung tumours. *Cancer Res.*, **49**, 6960.

HOWARD, E.W. & KNAUER, D.J. (1987). Characterization of the receptor for protease nexin-I: protease complexes on human fibroblasts. *J. Cell. Physiol.*, **131**, 276.

KESKI-OJA, J., BLASI, F., LEOF, E.B. & MOSES, H.L. (1988). Regulation of the synthesis and activity or urokinase plasminogen activator in A549 human lung carcinoma cells by transforming growth factor-$\beta$. *J. Cell Biol.*, **106**, 451.

LEE, H.-S. & WEINSTEIN, I.B. (1978). Epidermal growth factor, like phorbol esters, induces plasminogen activator in HeLa cells. *Nature*, **274**, 696.

MARKUS, G., TAKITA, H.K., CAMIOLO, S.M., CORASANTI, J.G., EVERS, J.L. & HOBIKA, G.H. (1980). Content and characterization of plasminogen activators in human lung tumours and normal lung tissue. *Cancer Res.*, **40**, 841.

MOLDENHAUER, G., MOMBURG, F., MÖLLER, P., SCHWARTZ, R. & HÄMMERLING, G.J. (1987). Epithelium-specific surface glycoprotein of Mr 34,000 is a widely distributed human carcinoma marker. *Br. J. Cancer*, **56**, 714.

MOMBURG, F., MOLDENHAUER, G., HÄMMERLING, G.J. & MÖLLER, P. (1987). Immunohistochemical study of the expression of a Mr 34,000 human epithelium-specific surface glycoprotein in normal and malignant tissues. *Cancer Res.*, **47**, 2883.

NEUMANN, C. & SORG, C. (1983). Regulation of plasminogen activator secretion, interferon induction and proliferation in murine macrophages. *Eur. J. Immunol.*, **13**, 143.

OSSOWSKI, L. & REICH, E. (1983). Antibodies to plasminogen activator inhibit human tumour metastasis. *Cell*, **35**, 611.

PEZZUTTO, A., DÖRKEN, B., FELLER, A. & 5 others (1986). HD37 monoclonal antibody: a useful reagent for further characterization of 'nonT/nonB' lymphoid malignancies. In *Leucocyte Typing II, Vol. 2*, Reinherz, E.L., Haynes, B.F., Nadler, L.M. & Bernstein, I.D. (eds), p. 391, Springer Verlag, New York.

PLOW, E.F., FREANEY, D.E., PLESCIA, J. & MILES, L.A. (1986). The plasminogen system and cell surfaces: evidence for plasminogen and urokinase receptors on the same cell type. *J. Cell Biol.*, **103**, 2411.

PYKE, C., KRISTENSEN, P., RALFKIAER, E. & 4 others (1991). Urokinase-type plasminogen activator is expressed in stromal cells and its receptor in cancer cells at invasive foci in human colon adenocarcinomas. *Am. J. Pathol.*, **138**, 1059.

ROLDAN, A.L., CUBELLIS, M.V., MASUCCI, M.T. & 5 others (1990). Cloning and expression of the receptor for human urokinase plasminogen activator, a central molecule in cell surface, plasmin dependent proteolysis. *EMBO J*, **9**, 467.

SCATCHARD, G. (1949). The attractions of proteins for small molecules and ions. *Ann. N Y Acad. Sci.*, **51**, 660.

SCHWARTZ, R., MOMBURG, F., MOLDENHAUER, G., DÖRKEN, B. & SCHIRRMACHER, V. (1985). Induction of HLA Class-II antigen expression on human carcinoma cell lines by IFN-gamma. *Int. J. Cancer*, **35**, 245.

STOPPELLI, M.P., CORTI, A., SOFFIENTINI, A., CASSANI, G., BLASI, F. & ASSOIAN, R.K. (1985). Differentiation-enhanced binding of the amino-terminal fragment of human urokinase plasminogen activator to a specific receptor on U937 monocytes. *Proc. Natl Acad. Sci. USA*, **82**, 4939.

STOPPELLI, M.P., TACCHETTI, C., CUBELLIS, M.V. & 5 others (1986). Autocrine saturation of pro-urokinase receptors on human A431 cells. *Cell*, **45**, 675.

TOWBIN, H., STAEHILIN, T. & GORDON, J. (1979). Electrophoretic transfer of proteins from polyacrylamide gels to nitrocellulose sheets: procedure and some applications. *Proc. Natl Acad. Sci. USA*, **76**, 4350.

VASSALLI, J.-D., BACCINO, D. & BELIN, D. (1985). A cellular binding site for the $M_r$ 55,000 form of the human plasminogen activator, urokinase. *J. Cell Biol.*, **100**, 86.

WORLD HEALTH ORGANIZATION. (1982). Histological typing of lung tumours. Second edition. *Am. J. Clin. Pathol.*, **77**, 123.